# Giant Ulcerated Colonic Lipoma in an Asymptomatic Patient

**DOI:** 10.7759/cureus.59400

**Published:** 2024-04-30

**Authors:** Madeline J Washburn, Christopher Johnson

**Affiliations:** 1 Internal Medicine, Baylor Scott & White Medical Center, Temple, USA; 2 Gastroenterology and Hepatology, Baylor Scott & White Medical Center, Temple, USA

**Keywords:** colon mass, screening colonoscopy, lipoma, colonic, ulcerated

## Abstract

Colonic lipomas are benign masses typically found incidentally during routine screening exams. They rarely grow large enough to become symptomatic. While most colonic lipomas are small and do not lead to complications, giant lipomas can present with symptoms ranging from changes in bowel patterns and mild abdominal pain to bowel obstruction. Ulcerated giant colonic lipomas are even more rare findings on screening colonoscopies. Diagnosis in this context can be challenging, and resection is warranted in most cases. Here, we describe an asymptomatic patient who presented for a screening colonoscopy and was found to have a giant ulcerated colonic lipoma.

## Introduction

Gastrointestinal tract lipomas are benign, submucosal tumors consisting of adipose tissue. Though data is limited, the incidence of colonic lipomas is thought to range from 0.2 to 4.4% [1]. Most colonic lipomas are found in the right hemicolon, though they have been reported in the descending and sigmoid colon in 25% of cases [2]. Lipomas are typically small, but those over 4 cm are considered giant [3] and can lead to intussusception and obstruction [2]. These patients can present with various symptoms, including abdominal pain, distension, and obstipation.

Most commonly, colonic lipomas are detected incidentally. They are usually smooth, round, and may or may not be connected by a stalk. Endoscopic signs include the naked fat sign, pillow sign, and tent sign [4]. As they continue to grow and evolve, giant colonic lipomas may develop ulcerations, necrosis, and other malignant-appearing characteristics. 

Here, we describe a case that demonstrates a rare and unusual presentation of a giant colonic lipoma in which the pathology report resulted in unanticipated benign findings. 

## Case presentation

A 62-year-old female presented for a routine screening colonoscopy. Her past medical history included chronic urinary tract infections and hemorrhoidectomy. Her colonoscopy eleven years ago was unremarkable. The patient had no family history of colorectal cancer and denied GI symptoms. 

**Figure 1:**
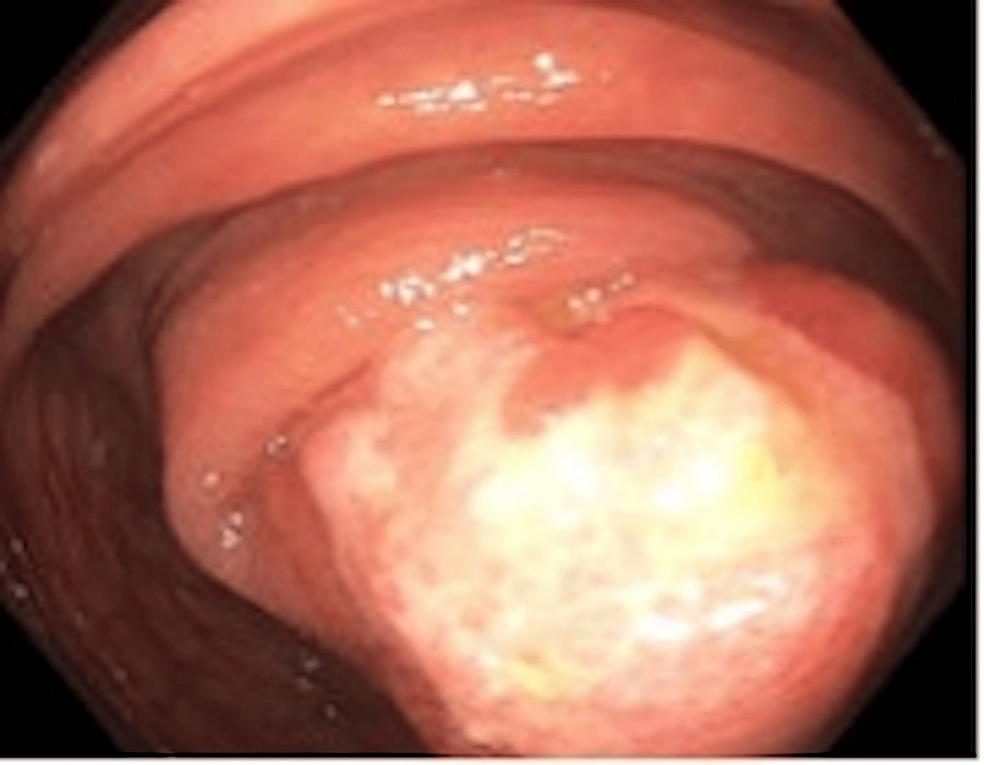
Malignant-appearing polyp at the hepatic flexure

**Figure 2:**
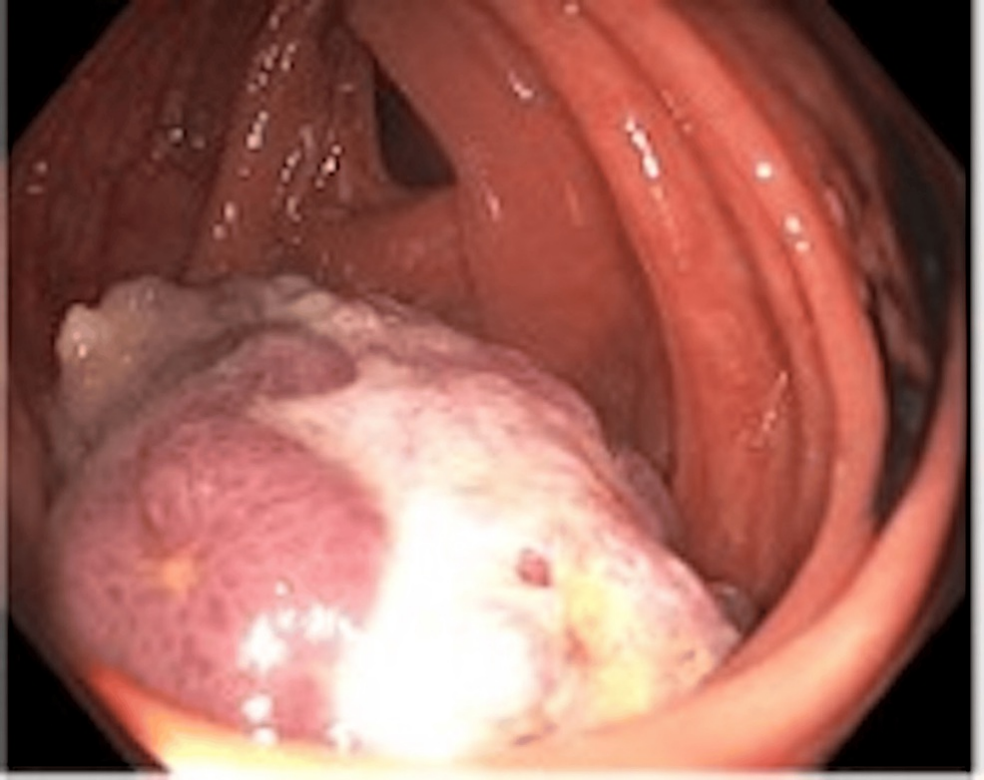
Malignant appearing polyp status-post polypectomy

Colonoscopy findings were significant for two polyps. The first was a 4-5 mm sessile polyp in the right colon that was removed and found to be a low-grade tubular adenoma. The second was a 5-cm pedunculated, ulcerated polyp near the hepatic flexure, shown in Figure 1; the endoscopic pillow sign was noted. The polyp was removed completely, as shown in Figure 2, by snare, using electrocautery, and retrieved intact with a Roth net. Two India Ink tattoos were placed distally. 

The patient presented to the Emergency Department the day after her procedure with a complaint of rectal bleeding. Her hemoglobin was within normal limits. She was admitted to the hospital, and a repeat colonoscopy showed ulceration at the hepatic flexure polypectomy site with no active bleeding. The polypectomy site was treated with a submucosal injection of epinephrine and placement of six hemoclips. The patient was discharged home and no further bleeding occurred. 

Histologic analysis of the large polyp showed mature adipose tissue consistent with lipoma with erosion and ulceration of the overlying mucosa. 

## Discussion

Colonic lipomas are the second most common benign masses found in the gastrointestinal tract behind adenomatous polyps [5]. Lipomas are composed of non-epithelial adipose tissue and are typically found in the submucosa of the large intestine. They are most commonly found in the right colon and are more common in women [6]. 

Colonic lipomas are typically diagnosed incidentally. Endoscopic features displayed by these masses include characteristics such as the tent sign, naked fat sign, or pillow sign. The tent sign occurs when the mass forms a tent shape while pulling at it during manipulation. The naked fat sign is seen when mature fat cells are visualized upon tunneled biopsy [7]. The pillow, or cushion, sign occurs when the mass returns to its previous shape after being released from forceps [2]. Given their fatty composition, these tumors may also be discovered through imaging modalities, such as computerized tomography and magnetic resonance imaging, as homogenous masses without solid components [7]. 

The risk of complications increases with the size of the lipoma. Small lipomas are typically asymptomatic, while approximately 75% of patients with giant lipomas will become symptomatic [6]. Symptoms are typically mild, but rarely, giant lipomas can lead to obstruction from intussusception with the lipoma as the obstructive lead point [2]. 

As lipomas grow, they can ulcerate and evolve into lesions mimicking malignancy, as was seen in our patient. Limited data estimate the total incidence of colonic lipomas is between 0.2 to 4.4% [1]. Ulceration of lipomatous tissues occurs when the exposed outer surface erodes. This may eventually lead to bleeding, crampy abdominal pain, melena, hematochezia, and potentially iron deficiency anemia. 

Expert opinions suggest treatment of most small colonic lipomas with endoscopic resection, while giant lipomas may warrant surgical intervention. However, in selected cases, endoscopic resection of giant lipomas may be the preferred method to prevent unnecessary surgery [8]. The proposed criteria for surgical management include lipomas larger than 4 cm with a sessile appearance or limited peduncle, lipomas presenting with severe symptoms such as intussusception, and those with involvement of the deeper mucosal layers [9]. Although our patient’s lipoma was greater than 4 cm, it had a clear, accessible stalk making our patient a reasonable candidate for endoscopic resection. 

## Conclusions

This case demonstrates how, regardless of ulceration, colonic lipomas can grow quite large without causing overt symptoms. Endoscopic resection of such lesions, in select cases, is a reasonable treatment strategy. 
